# Interventional radiology management of juvenile nasopharyngeal angiofibroma

**DOI:** 10.1016/j.radcr.2026.10.035

**Published:** 2026-11-07

**Authors:** Hailey Mangio, Casey Sun, L. Henry Bryant, Mary Arndt, Mustafa Al-Roubaie, Christopher Yeisley

**Affiliations:** a Morsani College of Medicine, University of South Florida, 560 Channelside Dr, Tampa, FL 33602, USA; b Department of Interventional Radiology, Moffitt Cancer Center, 12902 USF Magnolia Dr, Tampa, FL 33612, USA; c Department of Interventional Radiology, Naval Medical Center Portsmouth, 620 John Paul Jones Cir, Portsmouth, VA, 23708, USA

**Keywords:** Juvenile nasopharyngeal angiofibroma, Embolization, Interventional radiology, Polyethylene glycol microspheres

## Abstract

Juvenile nasopharyngeal angiofibroma is a rare, highly vascular tumor that occurs almost exclusively in adolescent and young adult males and is associated with substantial intraoperative bleeding risk during surgical resection. Preoperative intra-arterial embolization is commonly performed to reduce tumor vascularity and improve perioperative safety. We present a case of a 21-year-old male with a history of asthma presenting with persistent unilateral nasal congestion who was found to have a left-sided nasopharyngeal mass. Surgical pathology later confirmed sinonasal angiofibroma. Diagnostic cerebral angiography demonstrated tumor blush supplied by branches of the left sphenopalatine artery arising from the internal maxillary artery of the external carotid circulation without significant contribution from the internal carotid artery or ophthalmic artery anastomoses. Using a microcatheter system, selective catheterization of the left sphenopalatine artery was performed, and embolization was achieved with 200-micrometer polyethylene glycol microspheres until near stasis was observed. Postembolization angiography demonstrated marked reduction of tumor blush, consistent with successful devascularization. The patient subsequently underwent successful surgical resection with remarkably low intraoperative blood loss (estimated 50 mL). This case highlights the role of interventional radiology in targeted preoperative embolization for the management of juvenile nasopharyngeal angiofibroma. Selective embolization of the sphenopalatine arterial supply can significantly reduce tumor vascularity and facilitate safer surgical resection with minimal blood loss. The relatively insidious clinical presentation and use of polyethylene glycol microspheres, which, to our knowledge, have not been previously described in juvenile nasopharyngeal angiofibroma, represent distinctive clinical and technical features of this case.

## Introduction

Juvenile nasopharyngeal angiofibroma (JNA) is a rare, benign yet locally aggressive fibrovascular tumor of the posterior nasal cavity that occurs predominantly in adolescent males and commonly presents with unilateral nasal obstruction and recurrent epistaxis [1]. Despite its benign histology, JNA is clinically important because of its locally invasive growth pattern, potential for skull base extension, and marked hypervascularity, most often with supply from branches of the external carotid circulation [1].

Because of its characteristic vascularity and tendency for local invasion, imaging is central to JNA diagnosis and treatment planning. Computed tomography and magnetic resonance imaging are used for diagnosis and staging, while angiography further delineates feeder vessels, evaluates for internal carotid contributions or dangerous anastomoses, and guides endovascular management [[2], [3], [4]]. The mainstay of treatment for JNA is surgical excision; however, resection can be associated with substantial intraoperative hemorrhage because of the tumor’s marked vascularity [1]. Preoperative embolization is therefore commonly performed to reduce tumor blood supply and facilitate safer surgical removal [1].

Here, we present a case of JNA treated with selective preoperative embolization followed by surgical resection with remarkably low intraoperative blood loss. This case highlights the role of the interventional radiologist in identifying the tumor’s vascular supply, achieving effective and targeted devascularization, and facilitating safer operative management in a lesion classically associated with challenging intraoperative hemorrhage risk.

In addition to illustrating the multidisciplinary management of JNA, this case is notable for the relatively insidious 2-3 year clinical course in a 21-year-old patient with persistent unilateral congestion but no significant epistaxis or other prominent symptoms. The case also provides a technical example of embolization using polyethylene glycol (PEG) microspheres, which, to our knowledge, have not been previously described in the published JNA literature.

## Case report

A 21-year-old male presented with persistent unilateral nasal congestion and a recently identified left-sided nasopharyngeal mass. The patient reported unilateral nasal congestion for the past 2-3 years. His otolaryngologist identified a left-sided nasopharyngeal mass approximately one and a half month prior to presentation. He denied significant epistaxis or other symptoms related to the mass. His medical history was notable for asthma, for which he uses an inhaler. He does not smoke and rarely consumes alcohol. Surgical history included tonsillectomy and wisdom teeth extraction without anesthetic complications. Imaging demonstrated an angiofibroma, as shown in Fig. 1. Based on the extent of disease, the tumor was classified as Radkowski stage IIB. Preoperative embolization of the mass was coordinated with the interventional radiology team.

**Fig. 1:**
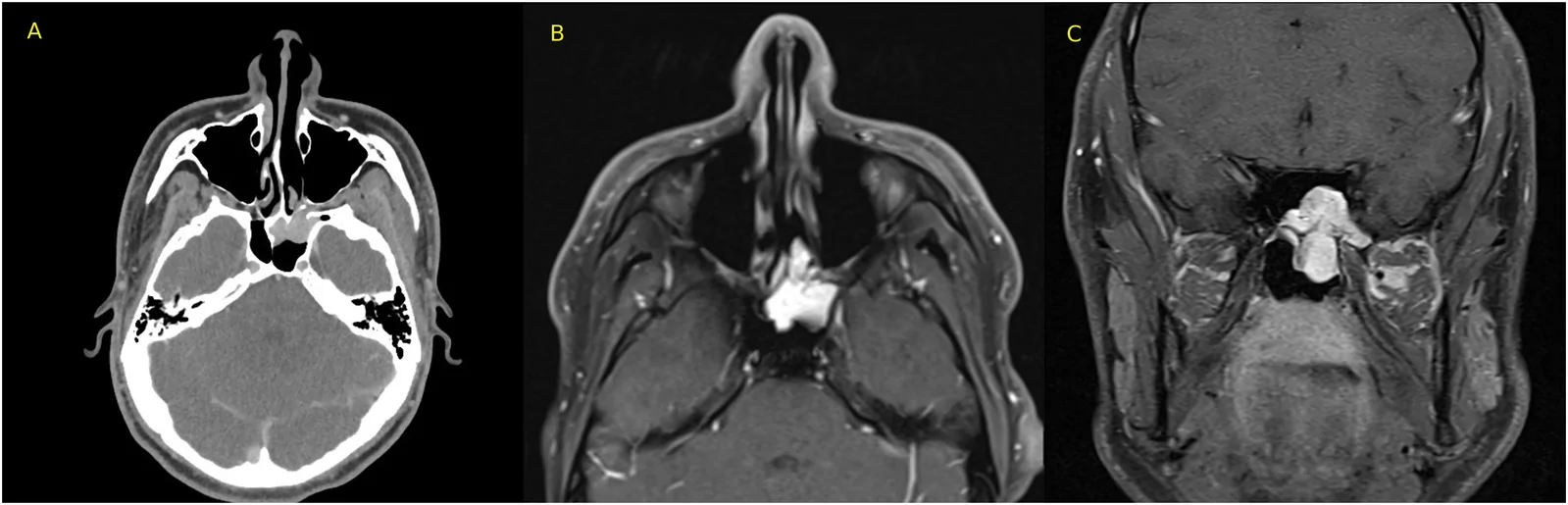
Contrast-enhanced axial computed tomography (CT) image of the brain shows a hyperenhancing invasive mass centered in the left sphenoid sinus (A). Axial and coronal T1 fat-saturated contrast-enhanced magnetic resonance imaging (MRI) demonstrate a homogeneously enhancing left sphenoid mass invading adjacent ethmoid air cells and nasopharynx, without intracranial involvement (B and C).

Angiographic runs of the right internal carotid artery (ICA), right external carotid artery (ECA), and left ICA did not demonstrate branch vessel supply to the tumor. Left posteroanterior (PA) and lateral ECA projection angiograms demonstrated a region of blush posterior to the ECA sphenopalatine artery branch, confirming it to be a contributing branch to the tangle of disorganized vessels making up the nasal angiofibroma, as seen in Fig. 2. No additional branches of the left ECA demonstrated supply to the tumor including the left lingual, facial, occipital, superficial temporal, internal maxillary, and middle meningeal arteries. No aneurysms, arteriovenous malformations, or fistulas were identified. There was no evidence of vasculopathy or vasculitis.

**Fig. 2:**
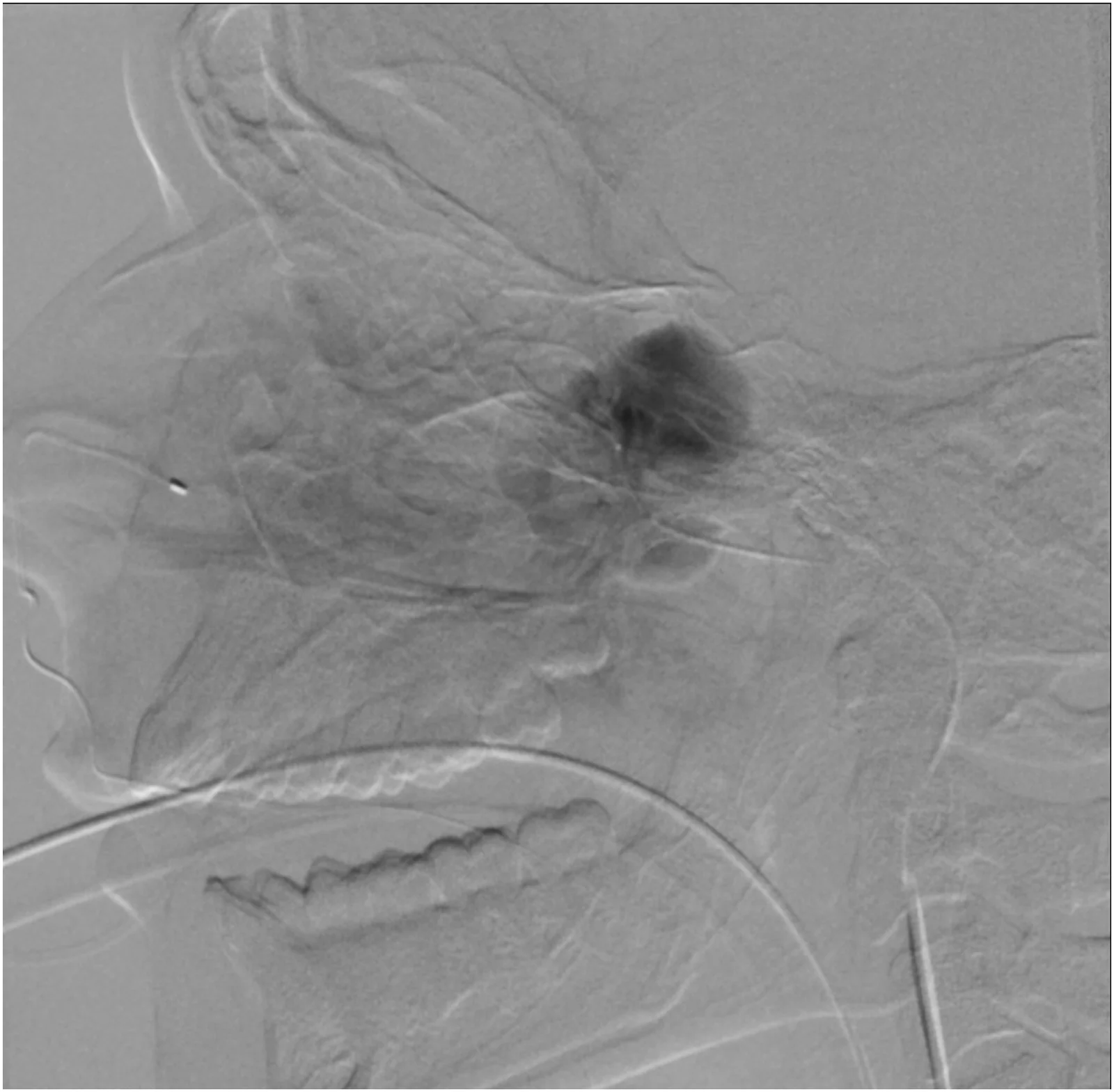
Delayed phase conventional angiogram of the left internal maxillary artery branch of the ECA demonstrates the contrast blush of the hypervascular nasopharyngeal mass.

Selective catheterization and angiography of the sphenopalatine artery branch of the left ECA was performed. Using a roadmap technique and the diagnostic catheter situated in the left ECA, the TRUSELECT microcatheter (Boston Scientific, Marlborough, MA) was advanced over an Aristotle 14 standard microwire (Scientia Vascular, West Valley City, UT) into the left internal maxillary artery. The microwire was then removed. PA and lateral projections demonstrated anterograde flow into the left sphenopalatine artery and its branches with the blush of the angiofibroma again visualized. No appreciable ophthalmic artery branches were evident. HydroPearl PEG particles (Terumo Interventional Systems, Somerset, NJ), 200 micrometers in size, were then injected until there was significant stasis of forward flow and subtle reflux around the catheter tip. Postembolization angiogram demonstrated decreased opacification of the angiofibroma blush consistent with embolization, as seen in Fig. 3. Postembolization left common carotid artery angiogram demonstrated no complications. The patient was then extubated and transferred to the intensive care unit in stable condition. He was transferred to the operating room the day following embolization for surgical resection of the mass.

**Fig. 3:**
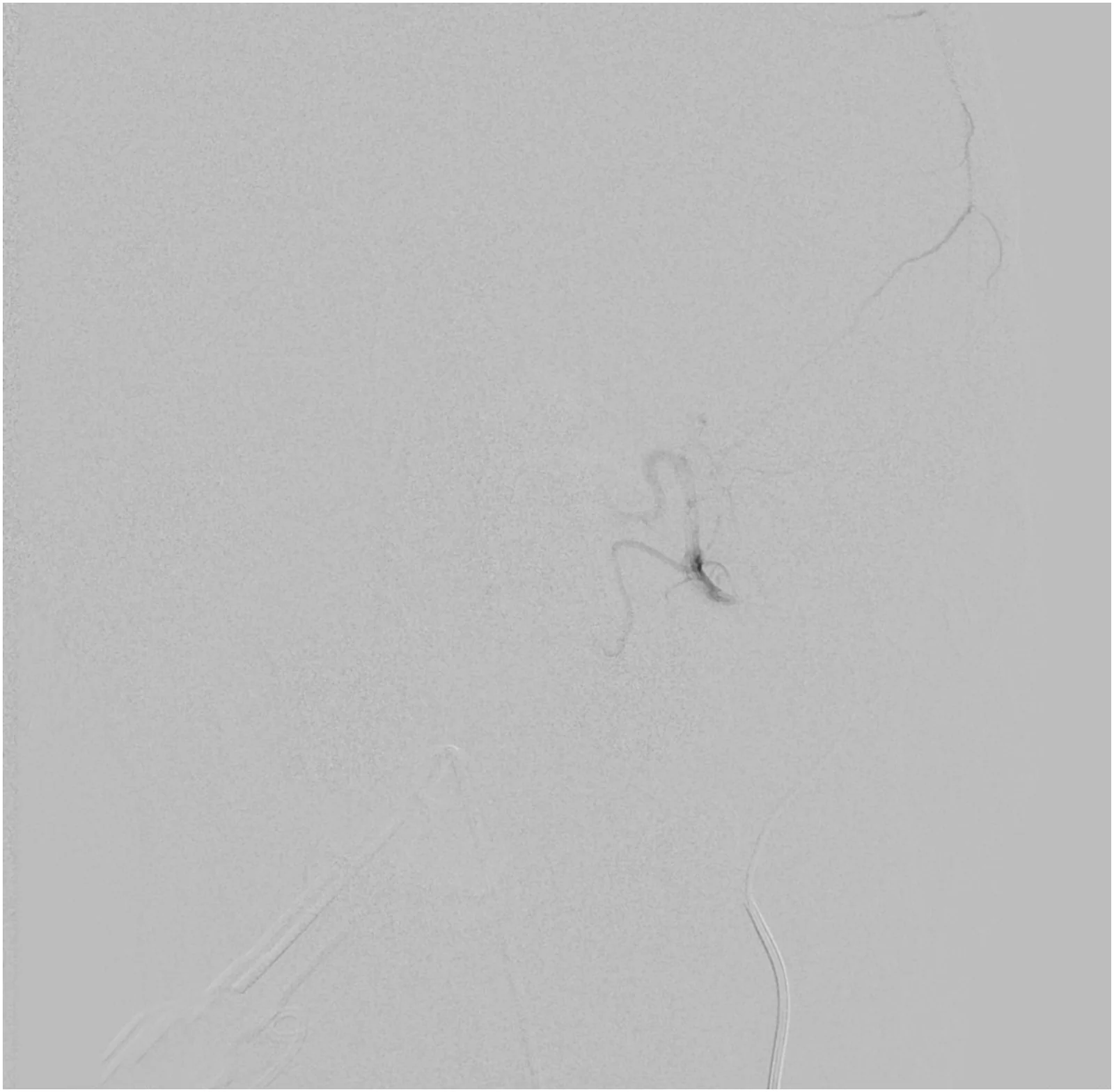
Postembolization conventional angiogram of the left internal maxillary artery shows no enhancement of the tumor.

Surgical resection was performed with an estimated blood loss of only 50 mL, which is notably very low. The specimen was sent for pathologic evaluation, presented in Fig. 4. Pathology indicated the presence of β-catenin highlights within stromal cells with appropriate control reactivity, further supporting the clinical diagnosis.

**Fig. 4:**
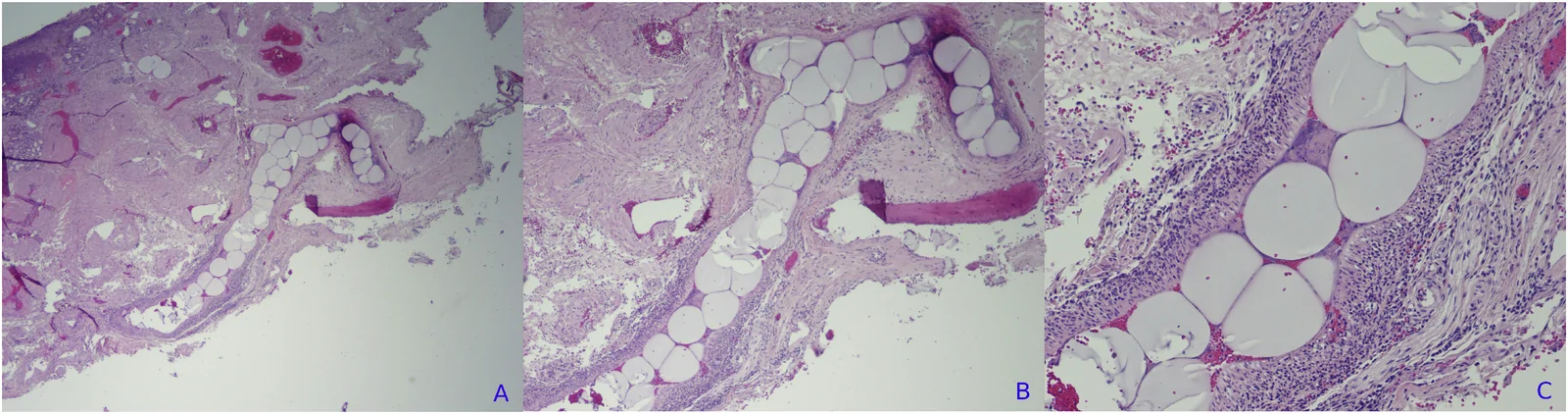
Biopsy of left sinonasal mass at 2× (A), 4× (B), and 10× (C) magnification demonstrates sinonasal tract angiofibroma with intravascular foreign material consistent with embolizing agent.

## Discussion

This case demonstrates the value of preoperative embolization in the management of JNA. In our patient, selective embolization of the sphenopalatine arterial supply with 200-micrometer PEG particles achieved marked devascularization of the tumor prior to surgery, and subsequent resection was associated with remarkably low intraoperative blood loss (50 mL). Given the pronounced vascularity that characterizes JNA, this outcome supports the practical role of interventional radiology in reducing operative hemorrhagic risk and facilitating safer definitive treatment.

The clinical presentation in this case was notable for both the patient’s slightly older age and the relatively insidious symptom course. JNA most commonly occurs in young males between 9 and 19 years of age and typically presents with unilateral nasal obstruction and recurrent epistaxis; symptoms are generally present for approximately 6 months to 1 year before diagnosis [5]. In contrast, our patient presented at age 21 after a 2- to 3-year history of persistent unilateral nasal obstruction without significant epistaxis or other prominent tumor-related symptoms. The prolonged symptom course, absence of epistaxis, and slightly older age at presentation represent distinguishing clinical features of this case.

Beyond the clinical presentation, the embolic material used in this case represents an additional distinguishing feature. Particulate embolization is an established approach for JNA, although the specific materials used vary considerably across the literature [6]. A 2026 meta-analysis of 40 studies including 448 patients found polyvinyl alcohol to be the most commonly reported embolic material (59.2%), followed by cyanoacrylate glue, gelatin sponge, ethylene vinyl alcohol copolymer, and coils; PEG microspheres were not represented among the reported embolic materials [6]. Prior reports of microsphere embolization for JNA are sparse and have described non-PEG microspheres [7,8]. PEG microspheres are nonresorbable, tightly calibrated, spherical particles with compressible properties, characteristics that may allow more distal occlusion and tighter packing within target vessels [9,10]. To our knowledge, PEG microspheres have not been previously described for JNA embolization in the published literature, making their use a technical contribution of this report.

Overall, the literature supports that preoperative embolization is a useful adjunct in JNA management and has been associated with low pooled embolization and surgical complication rates, although the literature remains heterogeneous with respect to embolization technique, material, and timing prior to surgery [11]. The outcome in our case is consistent with prior literature showing that preoperative embolization reduces blood loss during JNA resection. A 2023 systematic review and meta-analysis found that embolization decreases blood loss in JNA surgery, with mean intraoperative blood loss substantially lower in embolized patients than in those undergoing surgery alone (842 mL vs 1525 mL) [12]. In that context, the estimated blood loss of 50 mL in the present case is notably low. Greater operative blood loss has also been associated with tumor stage and lesions demonstrating combined external and internal carotid arterial supply rather than purely external carotid supply, underscoring the potential contribution of both tumor extent and vascular anatomy to operative risk [13]. However, more recent data suggests that with effective preoperative embolization, vascular complexity may not significantly affect resection outcomes [14].

These outcomes reflect the expected effect of preoperative devascularization in a hypervascular tumor. Preoperative embolization likely reduces blood loss during JNA resection by decreasing tumor vascularity, thereby limiting intraoperative hemorrhage and improving visualization of the operative field [15]. Preoperative angiography is also important in identifying dominant feeder vessels and vascular anatomy relevant to treatment planning [13].

Preoperative transarterial embolization is not without risk because extracranial-to-intracranial anastomoses may permit nontarget embolization into intracranial vessels, such as the ophthalmic artery [12]. Complications of nontarget embolization of intracranial vasculature can include stroke, blindness, and cranial nerve palsies [12]. Angiography is therefore important for assessing tumor vascular supply prior to embolization [5]. In the present case, angiography demonstrated no significant internal carotid arterial contribution to the tumor, and selective angiography of the sphenopalatine artery demonstrated no appreciable ophthalmic artery branches before particle delivery. Postembolization angiography demonstrated no procedural complications.

In conclusion, this case underscores the importance of selective preoperative embolization in minimizing blood loss during JNA resection and thereby reducing operative risk. It also highlights the value of interdisciplinary management of JNA, with interventional radiology serving a central role through angiographic mapping and targeted devascularization to facilitate safer resection of this characteristically hypervascular tumor. The relatively insidious clinical presentation and use of PEG microspheres, which, to our knowledge, have not been previously reported in the published JNA literature, provide additional clinical and technical context to the management of JNA by interventional radiology.

## Patient consent

Written informed consent for publication of this case report and associated images was obtained from the patient.
